# Experimental study on granite acoustic emission and micro-fracture behavior with combined compression and shear loading: phenomenon and mechanism

**DOI:** 10.1038/s41598-020-78137-0

**Published:** 2020-12-16

**Authors:** Yue Cao, Jinhai Xu, Liang Chen, Peng Wu, Faiz Shaikh

**Affiliations:** 1 School of Mines, China University of Mining and Technology, Xuzhou, China; 2 State Key Laboratory of Coal Resources and Safe Mining, China University of Mining and Technology, Xuzhou, China; 3 School of Mechanics and Civil Engineering, China University of Mining and Technology, Xuzhou, China; 4 School of Civil and Mechanical Engineering, Curtin University, Perth, Australia

**Keywords:** Energy science and technology, Engineering

## Abstract

One element that is essential to consider in underground mining engineering applications is the possibility of pillar failure, which can result in deadly geological disasters, including earthquakes and surface subsidence. Pillars are commonly present under an inclined state and are significantly dependent upon combined compression and shear loading. However, many scholars regard the pure uniaxial compression strength (UCS) of rock as the main evaluation index of pillar strength, which is inconsistent with the field practice. Hence, the present study developed a novel combined compression and shear test (C-CAST) system, which was applied in the investigative acoustic emission (AE) experiments to characterize the failure mechanism and micro-fracture behavior of granite specimens at different inclination angles. The experimental results presented the exponential decrease of UCS of inclined specimens with increase in the shear stress component. Changes in the inclination angle with a range of 0°–10° produced a splitting-shear failure fracture mode from the initial splitting failure. In comparison, an increase in the inclination angle from 10° to 20° produced a single shear failure fracture mode from the initial combined splitting-shear failure. The specimens exhibited nonlinearly reduced microcrack initiation (CI) and damage (CD) thresholds following an increase in the inclination angle, suggesting the dependence of the microcrack initiation and propagation on the shear stress component. The ratio of CI and CD thresholds to inclined UCS varies within a certain range, indicating that the ratio may be an inherent property of granite specimens and is not affected by external load conditions. Additionally, the rock fracture behavior was largely dependent upon the mechanism of shear stress component, as validated by the microcrack initiation and growth. Finally, a modified empirical formula for pillar strength is proposed to investigate the actual strength of inclined pillar. Results of a case study show that the modified formula can be better used to evaluate the stability of inclined pillars.

## Introduction

Preserving ore pillar is a popular method in underground mining engineering for meeting engineering requirements^1^. A sound pillar design is paramount to ensure the safety of mine workers while not compromising the productivity. Meanwhile, pillars are also the support systems of underground mining engineering, which can effectively control the surface subsidence. Recently, construction of underground reservoirs has become a hot topic in west of China, where pillars are often used as water barriers to prevent the loss of water resources. Hence, it is necessary to study the pillar stability for better engineering applications. Comprehensive understanding of the failure mechanism and micro-fracture behavior of pillar at varying conditions is necessary to develop the optimal pillar strength design^2–6^. Past literature focused on optimizing the experimental conditions as these significantly affected the rock material or rock-like material fracture behavior^7–13^. These studies reported the application of uniaxial compression or confined compression loading conditions. However, not enough studies investigated the fracture behavior of rock under combined compression and shear. This is essential to simulate the test conditions in line with the field conditions.

**Figure 1:**
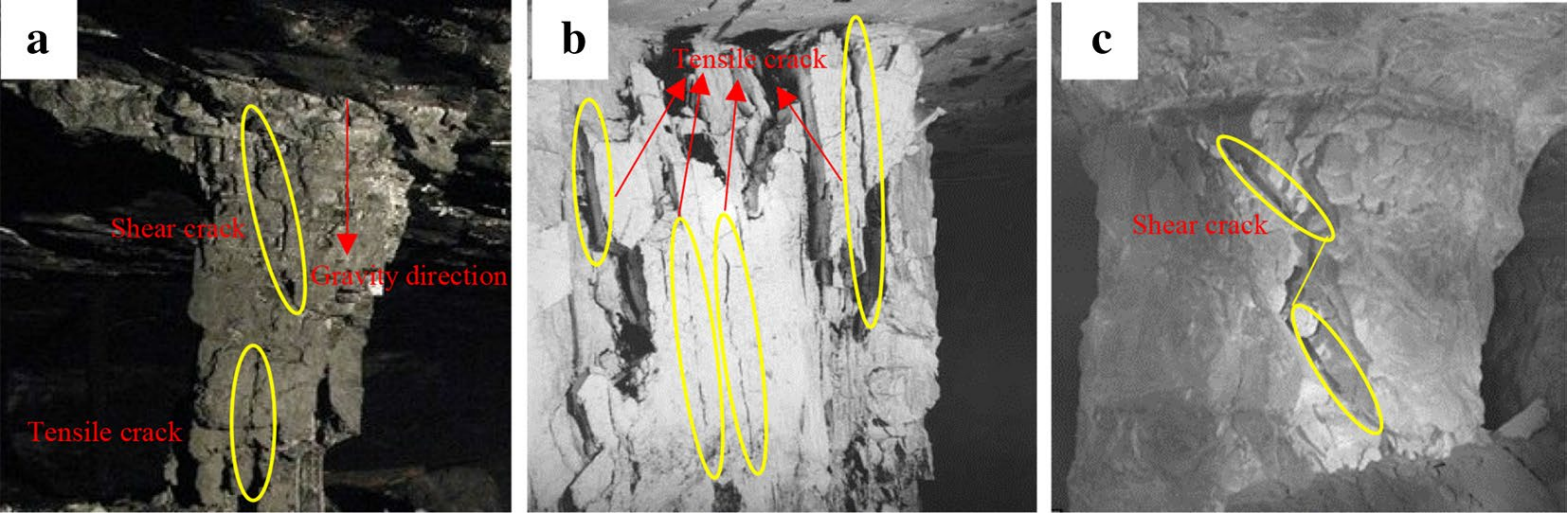
Engineering cases about pillars failure^1^. (**a**) Inclination limestone pillar; (**b**) Vertical gypsum pillar; (**c**) Low inclination stone pillar.

Many engineering applications have reported the importance of inclination angle as this significantly affects the pillar under combined compression and shear loading due to its relation to failure mode as shown in Fig. 1. Pariseau^14^ and Foroughi^15^ previously reported the effect of the external shear loading on coal pillar stability estimation in inclined coal seams. Jessu^16^ indicated the dependence of the pillar design on the inclination of ore body. Specifically, the pillar orientation exhibited axial loading (pure compression) when using horizontal pillars and oblique loading (combined compression-shear loading) when using inclined pillars. In addition, the shear stress component enhanced the pillar instability. Das^17^ observed the overestimation of inclined coal pillar strength using the traditional pillar strength formulae as these formulae do not consider the inclined rock strata shearing characteristics into account. In addition, the bedding plane shear failure also reportedly dominated the inclined rock strata. Ching^18^ suggested that the additional shear stress components may be a result of the inclined pillars instability as compared to the fat coal pillars. Suorineni^19^ challenged the use of the empirical pillar strength formulae to investigate the applicability of the conventional uniaxial compressive strength (UCS) given that this parameter only highlights the rock strength under pure compression. In comparison, their numerical simulation results indicated lower pillar strength due to increase in the pillar inclination. These findings validate the dependence of pillar stability on the pillar inclination. Therefore, characterization of the rock fracture behavior under combined compression and shear loading is essential to determine the inclined pillar instability mechanism.

Martin^20^ carried out pillar case studies, and found that the pillar strength is directly related to the pillar width-to-height ratio. Sheorey^21^ improved the calculation formula of coal pillar strength by introducing buried depth and width-to-height ratio. Ebrahim^22^ reported that weathering and moisture greatly reduce the strength of coal pillars. Esterhuizen^23^ stated that the destruction of the pillar is related to the spalling of the rock and the discontinuous shear planes of different angles in the pillar. Swift^24^ indicated that the increased weathering of the mine structure may lead to the destruction of the pillar. However, the above documents do not deal with the influence of the dip angle on the stability of the pillar, and ignored the relationship between the combined compression- shear load and the stability of the pillar. Xu and Dai^25^ applied a dynamic impact test, which implemented the modified split Hopkinson pressure bar (SHPB) system, to which the dynamic response and failure mechanism results in brittle failure of rocks were due to application of combined compression-shear loading. These rock or non-rock material were then subjected to combined compression-shear loading at a high strain rate. However, combined compression-shear loading of pillar in actual engineering practices necessitate long-term stability for optimal underground mining engineering construction applications. Therefore, characterization of the specimen properties and fracture behavior following the application of combined compression and shear at low strain rates remains a challenge.

The present study applied a novel combined compression and shear test (C-CAST) system to examine the standard granite specimen’s mechanical properties and fracture behavior under combined compression and shear loading at low strain rate. The acoustic emission (AE) technology is implemented to characterize the micro-crack initiation (CI) and micro-crack damage (CD) at various C-CAST inclination angles. Finally, a modified empirical pillar strength formula is proposed to investigate the actual strength of inclined pillar. A case study shows that the modified pillar strength formula can be better used to evaluate the stability of inclined pillars. The results can provide important reference for strength design and instability mechanism of inclined pillar.

## Results

### Effect of inclination of specimen on the failure patterns

**Figure 2:**
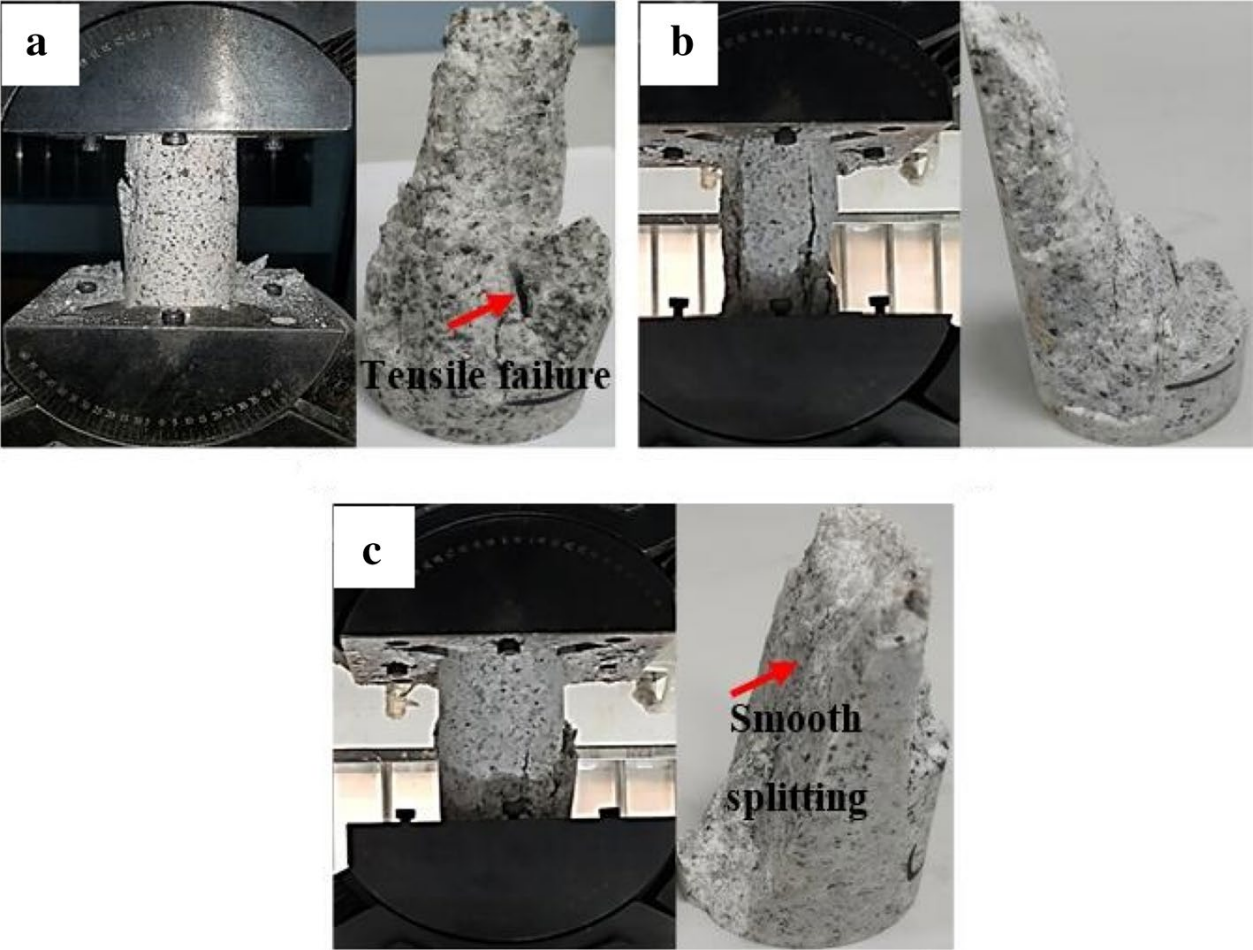
Granite specimen failure patterns under a uniaxial compression state: (**a**) Experimental case 1–1; (**b**) experimental case 1–2; and (**c**) experimental case 1–3.

Failure pattern of granite specimen under a pure uniaxial compression statein Fig. 2 shows a significant amount of parallel cracks formed on the specimen in axial direction, all of which clearly exhibit splitting failure. As a whole, the splitting plane is relatively smooth (see Fig. 2). Hence, the failure mode of granite specimens at 0° inclination is consistence with previous studies.

**Figure 3:**
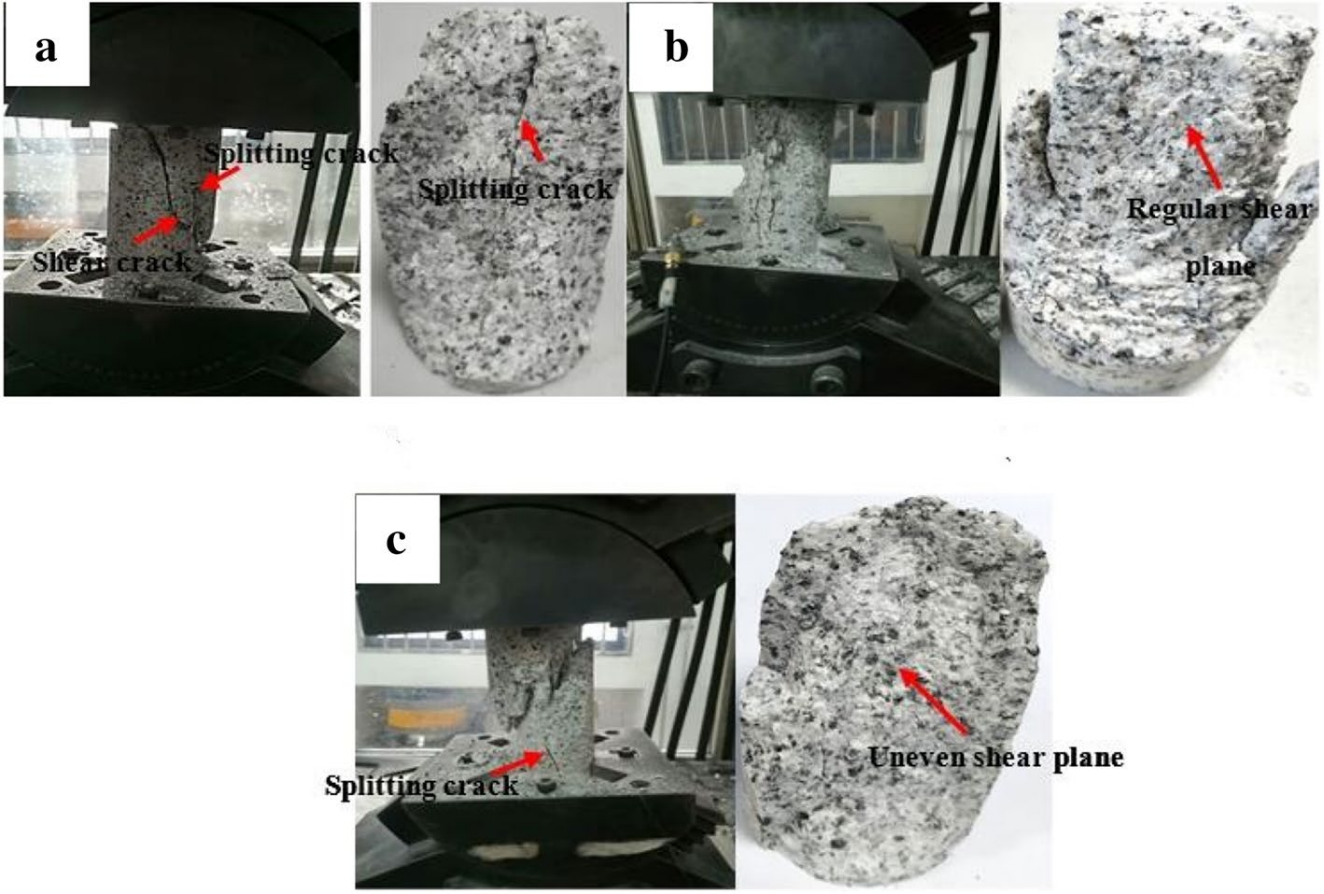
Granite specimen failure patterns at an inclination angle of 5°: (**a**) Experimental case 2–1; (**b**) experimental case 2–2; and (**c**) experimental case 2–3.

**Figure 4:**
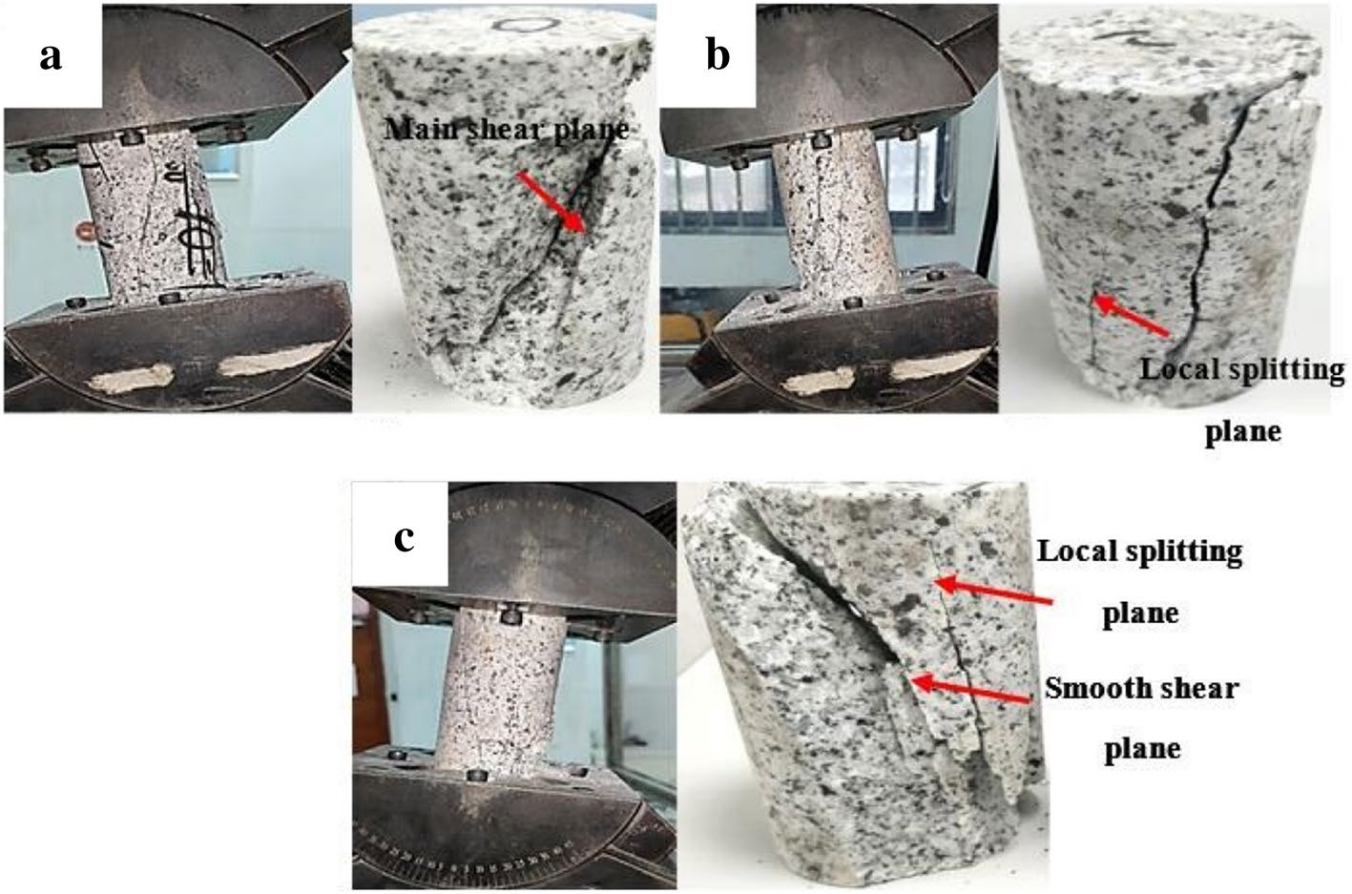
Granite specimen failure pattern at an inclination angle of 10°: (**a**) Experimental case 3–1; (**b**) experimental case 3–2; and (**c**) experimental case 3–3.

**Figure 5:**
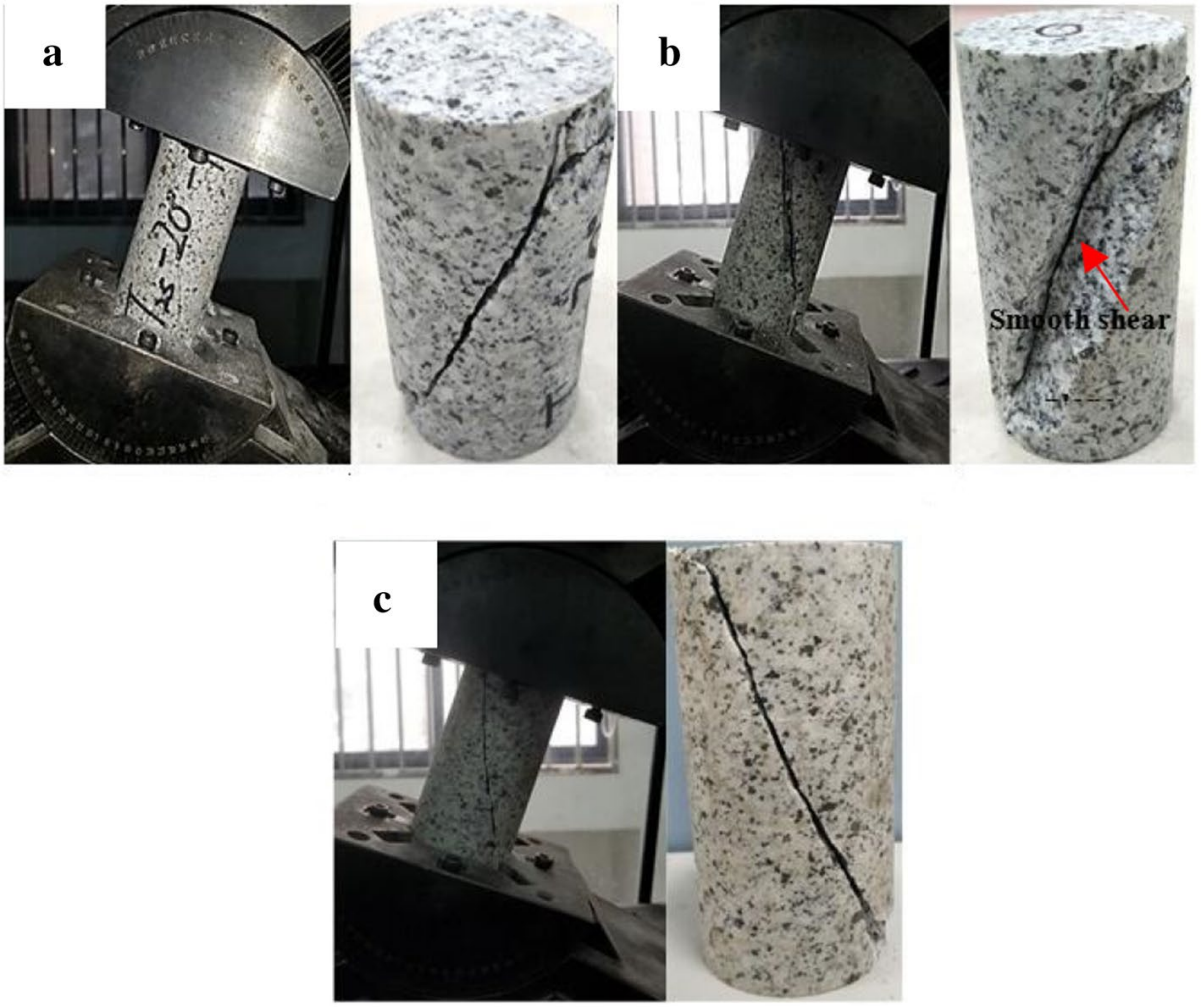
Granite specimen failure pattern at an inclination angle of 20°: (**a**) Experimental case 4–1; (**b**) experimental case 4–2; and (**c**) experimental case 4–3.

The failure pattern of the granite specimen at 5° inclination is shown in Fig. 3. According to Fig. 3a, two major cracks are formed on the specimen surface, one of which is nearly parallel to the specimen’s axial direction and the other is distributed at a certain angle from the axial direction. Both regular shear plane and uneven shear planes are alternately observed at an inclination angle of 5° (Fig. 3b,c), suggesting that the granite specimen failure mode is dependent on both the shear crack and tensile crack propagation. According to Fig. 4, upon reaching an inclination angle of 10°, almost all of the granite specimens show main shear failure plane from their upper and lower end faces. Some splitting cracks are also observed around the main shear plane (Fig. 4b,c). This shows that the shear failure tends to be more pronounced at 10° inclination compared to the 5° inclination. When the inclination angle further reaches 20° (Fig. 5), only one major shear crack runs through the specimen. As a whole, the fracture angle (i.e. the angle between macro-crack propagation direction and axial direction of specimen) gradually increases with increasing inclination angle from 10° to 20°. Hence, the results indicated that most significant single shear failure happens at an inclination angle of 20°.

According to the above results, the granite specimens show common splitting failure under traditional uniaxial compression due to the brittle failure of the hard rock. The combined splitting-shear failure mode dominated the specimen’s fracture at inclination angles of 5° and 10°. However, a granite specimen transition from combined splitting-shear failure to single shear failure is observed as the inclination angles increased from 10° to 20°, suggesting the presence of other intrinsic granite failure modes. Nevertheless, the failure modes are dependent upon the external loads^26–28^.

### Effect of inclination of specimen on peak strength

**Figure 6:**
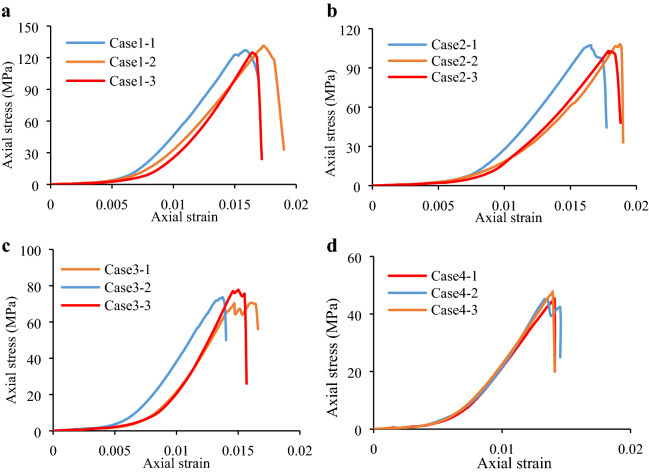
Granite specimen axial stress–strain curves at different inclination angles: (**a**) Under uniaxial compression; (**b**) 5°; (**c**) 10°; and (**d**) 20°.

Axial stress–strain curves at different inclination angles are generated using Eq. (13) and are shown in Fig. 6. The inclined uniaxial compression strength (IUCS) and peak shear strength ($\tau_{peak}$) of each specimen stress–strain curves are calculated based on Eqs. (13) and (14) (Table 1 and Table 2). It can be seen that the standard deviation of test results are 1.9–3.2 MPa with the coefficient of variation from 2.3 to 6.4% for IUCS and 0.2–1.1 MPa with the coefficient of variation from 2.2 to 6.7% for τ_peak_, respectively which indicate the reliability of the test data. With increasing inclination angle from 0° to 20°, the average IUCS is decreased by 82.7 MPa at a rate of 64.66%, while the average $\tau_{peak}$ is increased by 7.2 MPa at a rate of 77.42%.

**Table 1:** Granite specimen peak compression strengths at different inclination angles.

Experimental scenario (*θ*)	Experimental case	Strength (MPa)	Absolute deviation (MPa)	Relative deviation (%)	Mean value (MPa)	Standard deviation (MPa)	Coefficient of variation (%)
0°	1–1	127.2	0.7	0.5	127.9	3.2	2.5
	1–2	131.4	3.5	2.7			
	1–3	125.1	2.8	2.2			
5°	2–1	107.2	0.9	0.8	106.3	2.4	2.3
	2–2	108.1	1.8	1.7			
	2–3	103.6	2.7	2.5			
10°	3–1	70.6	2.1	2.9	72.7	1.9	2.6
	3–2	73.6	0.9	1.2			
	3–3	74.0	1.3	1.8			
20°	4–1	45.3	0.1	0.2	45.2	2.9	6.4
	4–2	42.3	2.9	6.4			
	4–3	48.0	2.8	6.2

**Table 2:** Peak shear strength of granite specimens at different inclination angles (peak shear strength was equal to zero at an inclination angle of 0°).

Experimental scenario (*θ*)	Experimental case	Strength (MPa)	Absolute deviation (MPa)	Relative deviation (%)	Mean Value (MPa)	Standard deviation (MPa)	Coefficient of variation (%)
5°	2–1	9.4	0.1	1.1	9.3	0.2	2.2
	2–2	9.5	0.2	2.1			
	2–3	9.1	0.2	2.2			
10°	3–1	12.5	0.4	3.2	12.9	0.3	2.3
	3–2	13.0	0.1	0.8			
	3–3	13.1	0.2	1.5			
20°	4–1	16.5	0.0	0.0	16.5	1.1	6.7
	4–2	15.4	0.9	5.8			
	4–3	17.5	1.0	5.7			

**Figure 7:**
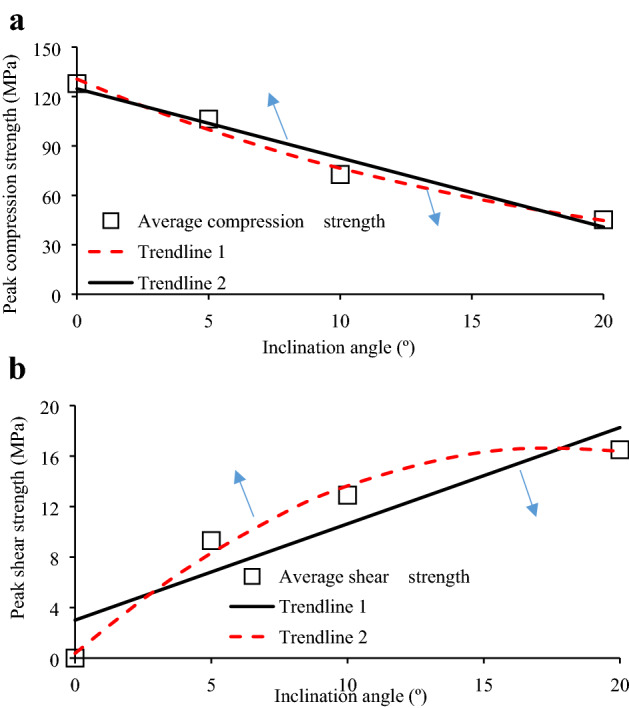
Specimen peak compression and peak shear strengths at different inclination angles. Trend lines 1 and 2 in Fig. 9a,b were fit against the data at an inclination angle range of 0° to 20°.

Figure 7 presents the dependence of the granite specimen inclination angle on the IUCS and $\tau_{peak}$, wherein trend lines 1 and 2 were applied for data fitting. The two fitting curves exhibited variances (*R*^2^) of 0.9656 and 0.9891 (Fig. 7a), respectively, suggesting exponential fitting of the IUCS at these inclination angles. The two other fitting curves in Fig. 7b exhibited variances (*R*^2^) of 0.989 and 0.8446, respectively, suggesting negative quadratic fitting between the inclination angle and $\tau_{peak}$ at an inclination range of 0° to 20°. A contribution coefficient of shear strength (CCSS) is proposed to determine the dependence of shear stress component on the mechanical properties.

1 $$\alpha = {{\tau_{peak} } \mathord{\left/ {\vphantom {{\tau_{peak} } {IUCS}}} \right. \kern-\nulldelimiterspace} {IUCS}}$$

where, *α* is the CCSS, which can be determined by the ratio of *τ*_peak_ to IUCS. Table 3 shows the CCSS of each granite specimen at various inclination angles. Notably, the CCSS was equal to zero at an inclination angle of 0°. The average CCSS exhibited about 8.91% and 18.74% increase from *θ* = 5° to *θ* = 10° and from *θ* = 10° to *θ* = 20° (Table 3), respectively. In comparison, the IUCS presented a gradual decrease, thus resulting in a weaker, shear stress-induced bond and friction strength among particles as well as reduced specimen deformation resistance.

**Table 3:** Contribution percentage of granite specimen shear strengths (α) at different inclination angles (contribution coefficient was equal to zero at an inclination angle of 0°).

Experimental scenario (*θ*)	Experimental case	Contribution coefficient (%)	Mean value (%)
5°	2–1	8.77	8.78
	2–2	8.79	
	2–3	8.78	
10°	3–1	17.71	17.69
	3–2	17.66	
	3–3	17.70	
20°	4–1	36.42	36.43
	4–2	36.41	
	4–3	36.46	

### CI and CD threshold determination techniques

**Figure 8:**
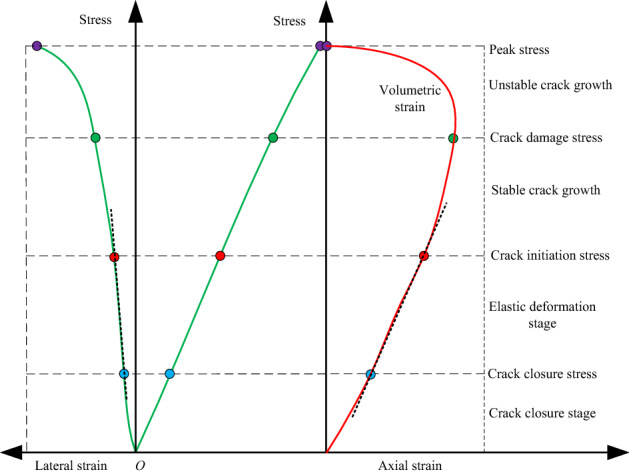
Brittle specimen critical stress thresholds and stress–strain curve schematic diagram following the addition of uniaxial compression^31^.

The strain energy release exhibited the crack initiation and propagation of rock material. In addition, the AE system was employed to monitor the elastic wave. Previous studies reported close relationship of the AE signal to the stress–strain curve, as well as its ability to effectively showcase the experimental crack evolution process of rock material^11,12^. Figure 8 presents the schematic diagram of the brittle specimen’s critical stress thresholds (i.e., CI and CD thresholds) and stress–strain curve characteristics following the application of uniaxial compression. Non-linear closure was present followed by a linear elastic stage, of which the stress–strain curve exhibited a linear slope. An increase in loading stress resulted in the development of new micro-cracks. In general, loading stress that induces new crack growth is termed crack initiation stress (*σ*_CI_), and it can be observed when the stress–volumetric strain and stress–lateral strain curves depart from linearity^28–30^. According to Brace^29^, the cracks are generally initiated at a stress level range of 0.3–0.5 UCS following the addition of pure uniaxial compression. Load application onto the specimen above the CI threshold then initiates the controllable and stable crack growth, wherein the fracture propagation is observed as a slow and stable process. As such, each crack growth increment requires the application of additional stress. Due to increase in load when the load reaches micro-crack damage threshold (*σ*_CD_), the crack propagation mode is out of control and begin to enter the stage of unstable crack growth. Hence, the threshold is the sign of unstable micro-crack propagation and coincides with the further drastic increase of the AE property rate.

The AE activity has the ability to accurately predict the CI and CD thresholds^9,12,28,30,32,33^. However, the threshold determination methods of CI and CD may vary, particularly for those with complex loading conditions. Wang ^11^reported the initial tensile crack stress level, which is generally triggered in the opening, as a potential CI threshold. Hu^30^ described two essential points: (1) *σ*_CI_: the cumulative AE count exhibits a significant increase; and (2) *σ*_CD_: the “cumulative AE hit vs. time” slope presents a dramatic increase following the CI threshold. To this, Zhao^31^ and Kim^34^ reported the applicability of the “cumulative AE energy or cumulative AE count vs. axial stress” slope change in properly defining the *σ*_CI_ and *σ*_CD_ of the rock material during loading. Alternatively, Eberhardt^35^ identified the ‘sharp points’ in the “AE property rate vs. axial stress” curve to determine the *σ*_CI_ and *σ*_CD_. The CI threshold is defined as the axial stress wherein the AE property rate exhibits a significant increase, and the point at which loading significantly changes the AE property rates prior to CI. The CD threshold is defined as the axial stress that induces a significant increase in the “cumulative AE property (i.e. cumulative AE counts and cumulative AE energy) vs. axial stress” slope curve following the CI threshold. Many scholars accept Eberhardt’s opinion that defined the two thresholds. Zhao^36^ showed that the AE hit line method can also be used to define the *σ*_CI_ and *σ*_CD_. Thus, the determination method of CI and CD thresholds is diverse.

**Figure 9:**
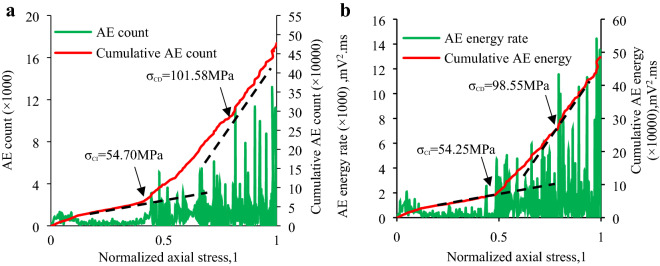
CI and CD thresholds of granite specimen in Test Case 1–1 (*θ* = 0°) determined by AE energy and AE hits: (**a**) CI and CD thresholds determined by AE count; (**b**) CI and CD thresholds determined by AE energy.

**Figure 10:**
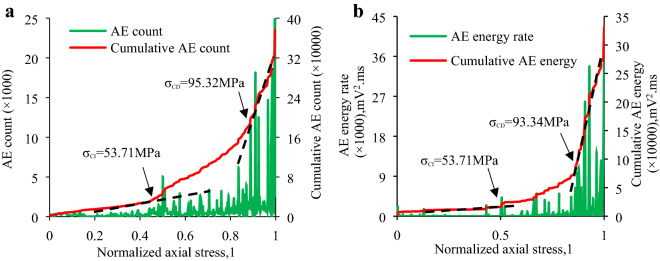
CI and CD thresholds of granite specimen in Test Case 2–2 (*θ* = 5°) determined by AE energy and AE count: (**a**) CI and CD thresholds determined by AE count; (**b**) CI and CD thresholds determined by AE energy.

**Figure 11:**
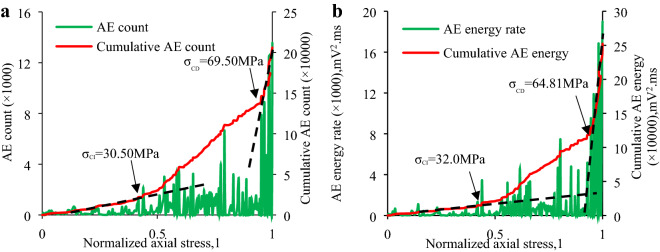
CI and CD thresholds of granite specimen in Test Case 3–3 (*θ* = 10°) determined by AE energy and AE count: (**a**) CI and CD thresholds determined by AE count; (**b**) CI and CD thresholds determined by AE energy.

**Figure 12:**
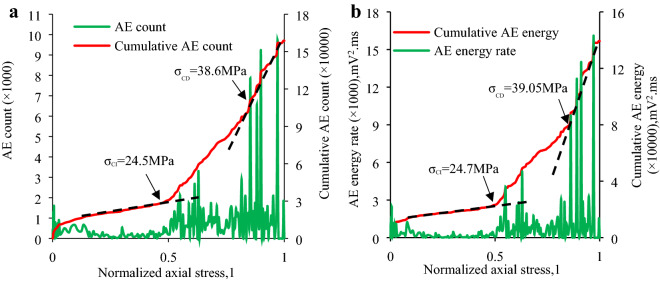
CI and CD thresholds of granite specimen in Test Case 4–1 (*θ* = 20°) determined by AE energy and AE count: (**a**) CI and CD thresholds determined by AE count; (**b**) CI and CD thresholds determined by AE energy.

In this section the *σ*_CI_ and *σ*_CD_ of the granite specimens at different inclination angles is established to examine the micro-fracture and damage behavior of the granite specimen via combining the above-mentioned methods, which primarily involve:(1) the sharp point of AE count rate and AE energy rate; and (2) the slope change of “cumulative AE counts and cumulative AE energy vs. axial stress” curve. Table 4 shows the *σ*_CI_ and *σ*_CD_ of each specimen determined by the AE count and their ratio to IUCS of the granite specimen at various inclination angles. Table 5 indicates the *σ*_CI_ and *σ*_CD_ determined by AE energy and their ratio to IUCS in the inclination range from 0° to 20°. Combining Table 4 and Table 5, the average *σ*_CI_ and *σ*_CD_ as well as the average *σ*_CI_/IUCS, *σ*_CD_/IUCS are obtained as shown in Table 6. Due to the IUCS and AE signals characteristic of the granite specimen in the same group are similar, the AE data of a part of laboratory results are presented in Figs. 9, 10, 11, 12, including the Test case1-1 (pure uniaxial compression), Test case2-2 (*θ* = 5°), Test case3-3 (*θ* = 10°) and Test case4-1 (*θ* = 20°).

**Table 4:** CI and CD thresholds determined by AE count and their ratio to corresponding peak compression strength of granite specimen at various inclination angles.

Experimental scenario (*θ*)	Experimental case	*σ*_CI_ (MPa)	Mean value (MPa)	*σ*_CD_ (MPa)	Mean value (MPa)	*σ*_CI_/IUCS (%)	*σ*_CD_/IUCS (%)
0°	1–1	54.70	53.30 ± 1.54	101.58	100.43 ± 3.16	41.67	78.52
	1–2	51.76		97.27			
	1–3	53.43		102.44			
5°	2–1	51.46	51.37 ± 2.44	92.50	94.09 ± 1.59	48.32	88.52
	2–2	53.71		95.32			
	2–3	48.93		94.46			
10°	3–1	34.52	32.23 ± 2.29	67.24	68.85 ± 1.61	44.34	94.70
	3–2	31.68		69.81			
	3–3	30.50		69.50			
20°	4–1	24.50	24.25 ± 1.56	38.60	38.79 ± 2.03	53.66	85.81
	4–2	22.69		40.82			
	4–3	25.57		36.94

**Table 5:** CI and CD thresholds determined by AE energy and their ratio to corresponding peak compression strength of granite specimen at various inclination angles.

Experimental scenario (*θ*)	Experimental case	*σ*_CI_ (MPa)	Mean value (MPa)	*σ*_CD_ (MPa)	Mean value (MPa)	*σ*_CI_/IUCS (%)	*σ*_CD_/IUCS (%)
0°	1–1	54.25	51.54 ± 3.24	98.55	99.31 ± 3.91	40.3	77.65
	1–2	52.07		96.17			
	1–3	48.30		103.22			
5°	2–1	51.82	50.69 ± 4.16	90.61	91.68 ± 1.66	47.68	86.25
	2–2	53.71		93.34			
	2–3	46.53		91.09			
10°	3–1	32.75	32.94 ± 1.14	65.47	65.83 ± 1.38	45.31	90.55
	3–2	34.08		67.21			
	3–3	32.00		64.81			
20°	4–1	24.70	24.59 ± 1.66	39.05	38.03 ± 1.75	54.40	84.14
	4–2	22.93		38.76			
	4–3	26.14		36.28

**Table 6:** Mean value of *σ*_CI_, *σ*_CD_, *σ*_CI_/IUCS and *σ*_CD_/IUCS determined by Tables 5 and 6.

Test scenario	CI threshold/*σ*_CI_, (MPa)	CD threshold/*σ*_CD_, (MPa)	*σ*_CI_/IUCS (%)	*σ*_CD_/IUCS (%)
*θ* = 0°	52.42	99.87	40.99	78.09
*θ* = 5°	51.03	92.89	48.00	87.39
*θ* = 10°	32.59	67.34	44.83	92.63
*θ* = 20°	24.42	38.41	54.03	84.98

### Effect of inclination effect of specimen on CI and CD thresholds

**Figure 13:**
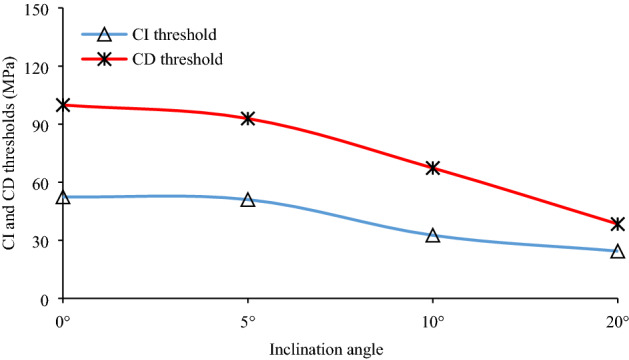
Specimen inclination angle with respect to the CI and CD thresholds.

Table 6 presents the CI and CD thresholds as well as average CI-to-IUCS and CD-to-IUCS ratios at different inclination angles for granite specimens. Figure 13 shows the CI and CD thresholds vs. inclination angles, wherein both thresholds present a gradual non-linear decrease with the increasing inclination angle. The inclination angles of 5°, 10°, and 20° exhibited reduction of average *σ*_CI_ values by 2.65%, 37.83%, and 53.41%, respectively, and reduction of *σ*_CD_ values by 6.99%, 32.57% and 61.54%, respectively, as compared to that with pure uniaxial compression. These results suggest the dependence of the rock material crack initiation and damage on the specimen’s inclination. In addition, the CD threshold of the specimen is first decreased slightly from 0° to 5° and then linear decreased sharply from 5° to 20°, indicating that once inclination angles exceed 5°, the other shear stress component more significantly affects the cracking and results in unstable micro-crack propagation.

The present work also calculated the *σ*_CI_ and *σ*_CD_ ratios to IUCS. The *σ*_CI_/IUCS ratio was within the range of 40.99% to 54.03% (Table 6). However, the ratio of *σ*_CD_/IUCS seems to be at a relatively high stress level of 78.09% to 92.63%, which is roughly consistence with that described by Bieniawski^28^ and Amann^9^ (0.7–0.9 UCS). This indicates that the test results of *σ*_CI_ and *σ*_CD_ are reliable. In general, it is possible for significant AE events to continue following the CD threshold, wherein the specimen first reaches its peak strength and subsequently exhibits fracture. Hence, in the field, we can monitor AE activities by using microseismical detector to determine whether the loading applied to pillar has reached the CD threshold and provide a precursor for pillar instability.

### Effect of inclination of specimen on the cumulative AE count and AE energy

**Figure 14:**
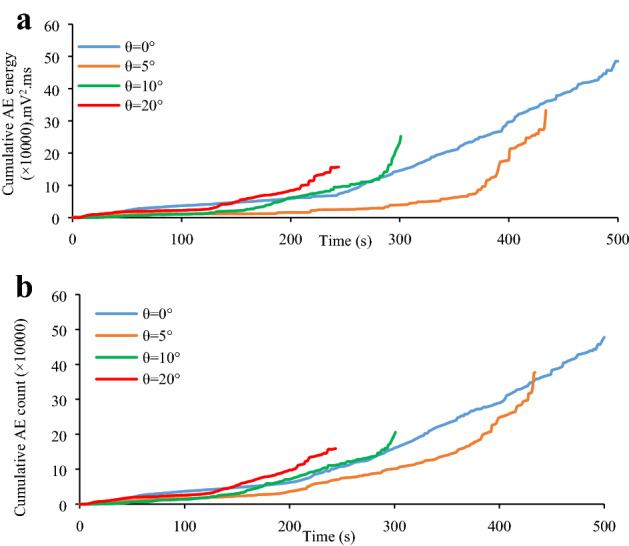
Specimen inclination angle with respect to the cumulative AE count and cumulative AE energy: (**a**) specimen inclination angle with respect to the cumulative AE energy; and (**b**) specimen inclination angle with respect to the cumulative AE count.

The relationship of the specimen inclination angle with the cumulative AE count and cumulative AE energy are characterized and is shown in Fig. 14. Similarly, the “cumulative AE properties (i.e., AE count and AE energy) vs. time” curve data at different inclination angles are also investigated (Figs. 9–12). The inclination angle significantly influenced the cumulative AE properties of granite specimen (Fig. 14). The granite specimen at inclination angles of 5°, 10°, and 20° exhibited a decrease in cumulative AE activity by 31.38%, 47.91%, and 67.63%, respectively and 21.05%, 56.87%, and 66.79%, respectively, for the cumulative AE count as compared to specimens with pure uniaxial compression. In addition, the required specimen failure loading time was inversely correlated to the inclination angle. The granite specimen exhibited applied loading times of 500 s for pure uniaxial compression (*θ* = 0°), 434 s for *θ* = 5°, 301 s for *θ* = 10°, and 244 s for *θ* = 20° inclination angles. These results suggest that the shear stress component enhanced the specimen fracture.

Starr^37^ related the average relative shear displacement ($U_{s}$) of the two crack surfaces with the driving shear stress ($S$), to which the following equation was derived:

2 $$U_{s} = {{\pi \left( {1 - \nu } \right)S \cdot c} \mathord{\left/ {\vphantom {{\pi \left( {1 - \nu } \right)S \cdot c} {2G}}} \right. \kern-\nulldelimiterspace} {2G}}$$

where $\nu$ and $G$ are Poisson’s ratio and shear modulus.$c$ is the crack length. Then, Starr further demonstrated that the reduction in elastic strain energy ($W_{e}$) during crack shear displacement is given by

3 $$W_{e} = {{\pi \left( {1 - \nu } \right)S^{2} \cdot c^{2} } \mathord{\left/ {\vphantom {{\pi \left( {1 - \nu } \right)S^{2} \cdot c^{2} } {2G}}} \right. \kern-\nulldelimiterspace} {2G}}$$

According to Eq. (3), the reduced elastic strain energy was proportional to the square of the driving shear. stress, indicating that a gradual decrease in the AE energy following an increase in the shear stress would be observed due to the change in elastic strain energy. This assumption was consistent with the experimental results, suggesting its validity. In addition, the higher shear stress enhanced the specimen damage. A gradual increase was observed in the shear stress component at higher inclination angles. As a result, the specimen was more likely to achieve its peak strength at a shorter time following the application of the same stress loading as well as with combined compression and shear loading as compared to specimens with pure uniaxial compression. This is consistent with the results shown in Fig. 14.

## Discussion

Many scholars have investigated the failure modes of granite specimens collected from different regions under pure uniaxial compression^38–41^. Their results shown that the granite mainly presents splitting failure mode with the tensile cracks propagation at room temperature. Bieniawski^28^ suggested the use of the modified Griffith failure criterion to characterize the crack initiation mode of brittle rock, especially for granite failure with inclined uniaxial compression. This criterion manifests distribution spalling fractures sub-parallel against the maximum principal stress direction^29,32,33,42,43^. The maximum principal stress was parallel to the axial direction with pure uniaxial compression, thereby allowing splitting failure. However, higher inclination angles gradually produced higher shear stresses, thereby generating deviations in the maximum principal stress direction from the axial specimen direction. Hence, the shear failure tends to increase with increasing inclination angle, as shown in Fig. 1.

The evolution process of rock fracture was dependent upon the random micro-crack distribution and particle friction resistance^44^. Higher shear stresses reduced the particle friction resistance and enhanced the micro-crack initiation and propagation^26^. The applied shear stress on the particle surface with pure uniaxial compression was significantly dependent upon the local stress state redistribution. Hence, it requires greater loading force to realize the stress adjustment inside the specimen. However, in the IUCS test, the shear stress component can be directly generated by the specimen inclination condition. In addition, higher inclination angles produced higher shear stresses, thus further enhancing micro-crack slippage as well as micro-crack initiation and propagation even at lower axial loads. Overall, this phenomenon gradually decreased the IUCS at higher inclination angles.

Previous reports undermined the UCS of rock material as these are dependent upon external loading^16,26,27^. In general, UCS is the primary reference index employed for pillar strength designs based on experimental tests performed under pure uniaxial compression conditions. However, field applications have indicated that multiple pillars exhibit inclination angles and loadings due to combined compression and shear loading^17^. Whereas, the evaluation methods of pillar strength based on traditional UCS test ignore the influence of inclination angle to the pillar stability. It seems unreasonable and limited. Hence, this paper suggests that the corrected inclined UCS should be used to design the pillar strength reasonably based on the actual situation.

Tapponier and Brace^32^ indicated that micro-crack extension at the crack initiation stress point basically can only be characterized at the grain-scale. Nevertheless, higher shear stresses can lower the particle friction resistance^26^, indicating that the shear stress can accelerate the crack initiation of the material. In addition, Alkan^45^ also observed that the new crack occurs and original crack reopens after elastic deformation with increasing shear stress, which indicate the important of shear stress on the *σ*_CI_. Generally, when the loading stress reaches the *σ*_CI_, the rock specimen still maintains a good bearing capacity, whereas the growth of micro-crack start to stable, resulting in the increased damage of the specimen^31,34,36^. Hence, CI threshold may be used as the initial damage point to judge whether the pillar has entered the stable cumulative damage state.

Nicksiar and Martin^46^ investigated low porosity crystalline crack propagation of specimens using a pure uniaxial compression test, of which the experimental results indicated tensile cracking dominance at the initial loading stages. In comparison, shear crack was exhibited following CI, and it was mainly observed at values close to the peak strength. It means that the shear crack can aggravate the unstable damage degree of the specimen. Brace and Byerlee^47^ investigated the sliding friction effect of rock material at micro-crack surfaces and found that the friction coefficient of micro-crack mainly dependent upon the sliding-induced extent of the wear. Nevertheless, the specimen sliding friction was highly dependent upon the shear stress^26^, thereby subsequently increases the shear crack. A gradually lowered CD threshold was observed at higher inclination angles following the addition of combined compression and shear loading as compared to the specimens with pure uniaxial compression.

Previous studies have reported many pillar stability and design methods, all of which apply Lunder’s “Confinement Formula”^48^ to calculate the pillar strength:

4 $$P_{s} = K \cdot UCS \cdot (C_{1} + C_{2} \kappa )$$

where $P_{s}$ is pillar strength;$K$ is pillar strength size factor;$C_{1}$ and $C_{2}$ are empirical rock mass constants;$\kappa$ is pillar friction term, which can be written as:

5 $$\kappa = \tan \cdot \{ \cos .^{ - 1} [(1 - Cpav)/(1 + Cpav).]\}$$

6 $$Cpav = Coeff * [\log (\omega + 0.75)]^{{{{1.4} \mathord{\left/ {\vphantom {{1.4} \omega }} \right. \kern-\nulldelimiterspace} \omega }}}$$

where $Cpav$ is average pillar confinement; $Coeff$ is coefficient of pillar confinement; $\omega$ is the ratio of pillar width to height.

According to Eq. (4), it can be seen that the pillar strength is closely related to the UCS of rock material. Then, the strength loss factor (α) is introduced to obtain the relationship between UCS and IUCS as follows:

7 $$\alpha \cdot \left( \theta \right) = {{(UCS - IUCS)} \mathord{\left/ {\vphantom {{(UCS - IUCS)} {UCS}}} \right. \kern-\nulldelimiterspace} {UCS}}$$

where $0 \le \alpha \le 1$. Integrated Eq. (7), The following Equation will be deduced as

8 $$IUCS = UCS \cdot (1 - \alpha )$$

**Figure 15:**
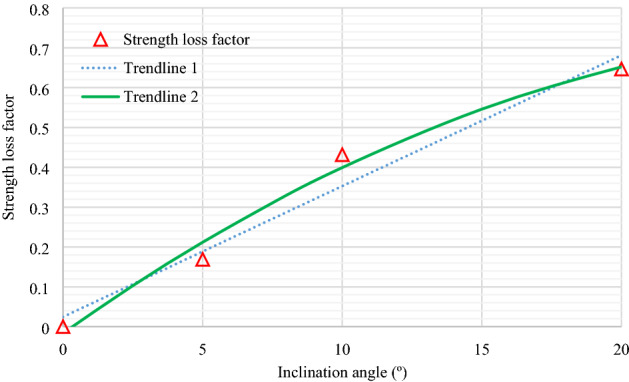
Effect of inclination angles on strength loss factor. Trendline 1 and Trendline 2 in Fig. 15 was fit against the data at an inclination angle range of 0° to 20°.

When $\alpha = 0$, IUCS is equal to UCS. With the strength loss factor increases, IUCS gradually decrease. According to the Table 1 in the paper, we can obtain the relationship between $\alpha$ and $\theta$, as shown in Fig. 15. In addition, the linear and quadratic functions are used to fit this data.

From the Fig. 15, the strength loss factor can also be rewritten as follows:

9 $$\alpha \cdot \left( \theta \right) = A_{0} + A_{1} \theta + A_{2} \theta^{2} \ldots A_{i - 1} \theta^{i - 1} + A_{i} \theta^{i} = \sum\limits_{i = 0}^{n} {A_{n} \theta^{n} }$$

where *A*_*i*_ defines the fitting coefficient, which is experimentally determined. Substituting Eqs. (8) and (9) into Eq. (4), two modified pillar strength formula considering the effect of pillar inclination angle can be obtained as follows

10 $$P_{s} = K \cdot UCS \cdot \left( {1 - \sum\limits_{i = 0}^{n} {A_{n} \theta^{n} } } \right) \cdot (C_{1} + C_{2} \kappa )$$

Let’s assume that there is a granite pillar with the ratio of pillar width to height of 2.0. A certain parameter in Eq. (10) was first defined to calculate the pillar strength. In general, $Coeff$ had a value of 0.45, which is suggested to generate extraction ratios ranging between 80 to 85% ^49^. Hence, the values of $Cpav$ in Eq. (6) and $\kappa$ in Eq. (7) can be deduced, respectively, which are 0.25 and 2.67. A previous study defined the values of $C_{1}$ and $C_{2}$ as 0.68 and 0.52, respectively. The pillar strength size factor had a value of 0.5 based on the shape effect formula^48^. The UCS of granite sample at 0° inclination is 127.9 MPa, as shown in Table 1.

Table 7 shows the pillar strength calculated by Eq. (10) under different inclination angles. From the Table 7, we can see that the pillar strength is approximately 52.63 MPa for 0° inclination when the strength loss factor satisfies the quadratic function (see Fig. 15). For 10 º and 20 º inclination, the actual inclined pillar strength was only 0.59-fold (31.03 MPa) and 0.34-fold (17.72 MPa) that of the horizontal pillar, respectively. Therefore, the strength of the pillar is easily overestimated if ignoring the influence of inclination angle. It means that the modified pillar strength formula can be better used to evaluate the stability of inclined pillars.

**Table 7:** Pillar strength under different inclination angles.

*θ* (°)	0	5	10	15	20
Quadratic function (MPa)	52.63	40.80	31.03	23.34	17.72
Linear function (MPa)	50.53	42.03	33.54	25.04	16.54

Higher inclination angle altered the initial granite specimen splitting failure (*θ* = 0°) to the combined splitting-shear failure (*θ* = 5° and 10°) to the single shear failure (*θ* = 20°), indicating that splitting failure is not the inherent attributes of hard rock and can be affected by external loading. The peak compression strength of the inclined specimen decreases almost exponentially with increasing inclination angle. Hence, the corrected inclined uniaxial compressive strength (UCS) should be recommended to be incorporated in empirical pillar strength formulae for designing the pillar strength reasonably.

The granite specimen’s CI and CD thresholds exhibited a gradual decrease at higher inclination angle, suggesting strong dependence of the shear stress on the combined compression and shear loading, which can weaken the frictional resistance among particles and accelerate the micro-crack initiation and damage especially at large inclination angle. In addition, the CD threshold first decreases slightly from 0° to 5° and then linear decreases sharply from 5° to 20°, indicating that once inclination angle exceeds 5°, the effect of additional shear stress component on the unstable propagation of micro-crack is more significant.

The contribution coefficient of nonlinear increase of shear stress with increasing inclination angles shows that the changes in the CI and CD threshold-to-peak compression strength ratios are proportional to the range of 40.99–54.03% for CI and 78.09–92.63% for CD. These results nullified the effect of the ratio on the specimen inclination angle.

The inclination angle is significantly dependent upon the AE properties (*i.e*., cumulative AE count, cumulative AE energy, and AE duration). Lowered AE properties are observed at higher inclination angles as compared to the pure uniaxial compression specimens. This is mainly because the shear stress is beneficial to the slipping of micro-crack surface and reduce the release of elastic strain energy, which further leads to a decrease in cumulative AE properties.

The present work reported a modified empirical pillar strength formula to characterize the actual inclined pillar strength. According to the case results, the inclined pillar exhibited a lower bearing strength as compared to the horizontal pillar. These results validated the applicability of the modified pillar strength formula in evaluating inclined pillar stability.

## Methods

### Experimental material

**Figure 16:**
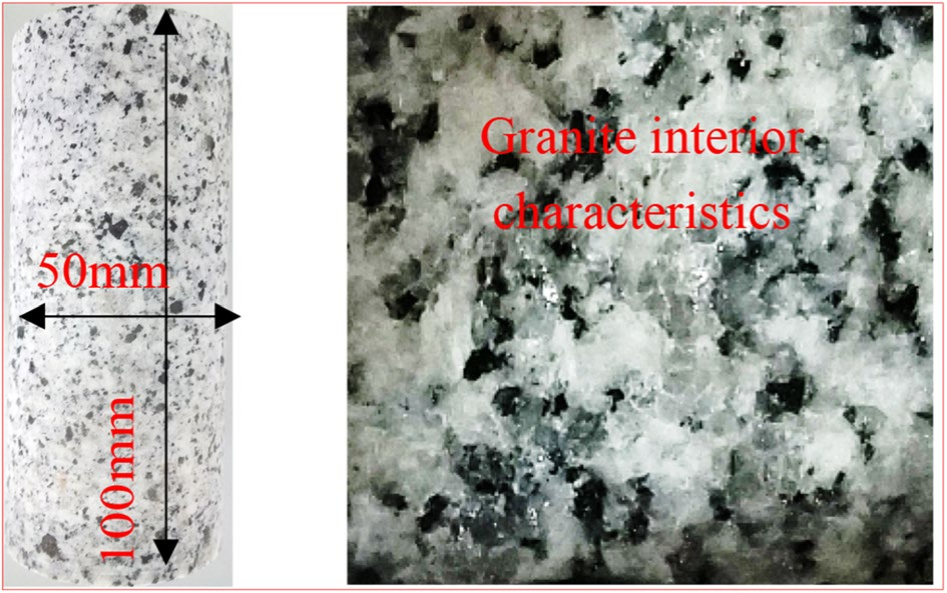
Interior characteristic of the granite studied in this work.

The presented experiments employed granite collected from Linyi City, Shandong Province, China. Ultrasonic velocity measurements were performed at each direction, to which the consistent 4,350 m/s results at a natural density of approximately 2730 kg/m^3^ exhibited good isotropy. The employed granite material had a crystalline and blocky structure, such that a light gray color was observed at the specimen surface (Fig. 16). The drilled cylindrical granite specimens had diameters of 50 mm and heights of 100 mm, all of which were cut to appropriate lengths (Fig. 16). These specimens had a height-to-width ratio of 2.0. The granite specimen fulfilled the International Society for Rock Mechanics (ISRM) size requirement. The cylindrical specimen had a surface roughness of less than 0.02 mm as well as end surfaces, which were perpendicular to its axis, of less than 0.001 radians to ensure a flat and smooth specimen surface^50^.

### C-CAST and AE monitoring system

**Figure 17:**
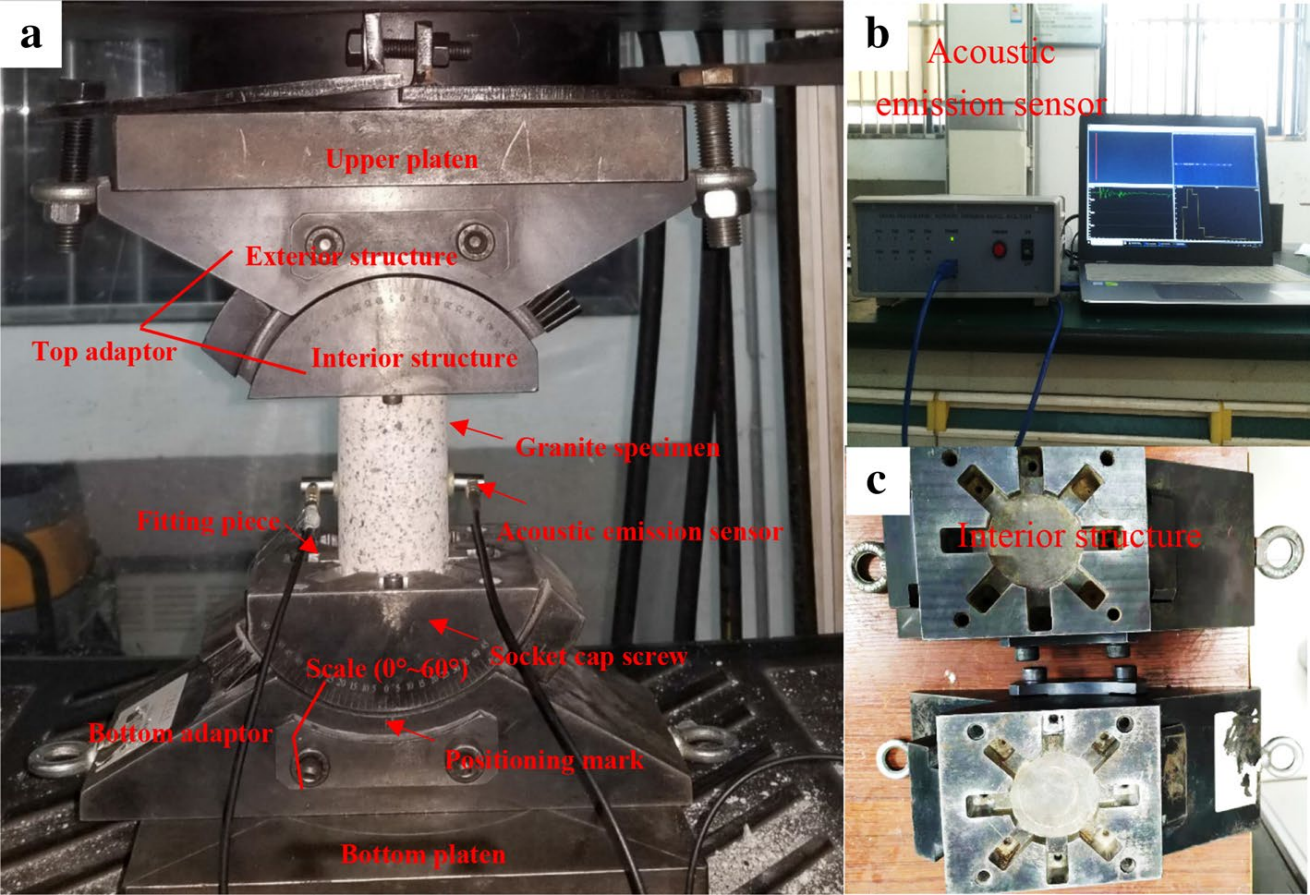
C-CAST system adapter and its specimen end connection.

To examine the dependence of the combined compression and shear loading on the granite strength properties and fracture behavior at low strain rate, the present work generated a novel C-CAST system. This C-CAST system, which is composed of two symmetrical adaptors, termed the top adaptor and bottom adaptor (Fig. 17). Each adaptor mainly includes interior structure and external structure. Each external adaptor structure has its positioning mark on the material testing system (MTS) platen. The interior structure of each adaptor is engraved with dials, which can accurately determine the inclination angle of the specimen and avoids the error caused by manual measurement. The granite specimen is situated between the upper platen and bottom platen. The test specimens are divided into four groups, corresponding to inclination angles of 0°, 5°, 10°, and 20°, of which each group has three specimens. All C-CAST system uniaxial compression experimental tests are conducted at the China University of Mining and Technology, China. All applied granite specimen load and displacement values were simultaneously performed and recorded at data collection intervals of 0.1 s until failure is observed. Additionally, the stress loading experiments are conducted at a loading rate of 0.5 MPa/s. If the test results by the specimens of each group had a large discreteness, the supplementary tests are carried out to reduce the experimental error.

All AE signals that are dependent upon the sudden growth of micro-cracks or slip along existing crack surfaces are characterized using a DS5 full-information AE measurement system with an 8-channel transient-recorder. Two adhesive-affixed sensors onto the specimen surface are employed to detect these AE events (Fig. 17), wherein the center frequency is set at 150 kHz. The threshold is the lowest value of the signal collected by the acoustic emission equipment. Its setting can effectively reduce the influence of the background noise of the experimental site on the experimental parameters. The acoustic emission threshold of this experiment is 40 dB^31,36^. Additionally, the AE system maintained data acquisition-MTS synchronization to ensure the accuracy of AE data. Following the completion of the tests, the granite specimens CI and CD thresholds are analyzed using the combined AE count and AE energy, which presented the dependence of the inclination angle on the specimen micro fracture behavior.

### Calculation methods of compression and shear stress components

**Figure 18:**
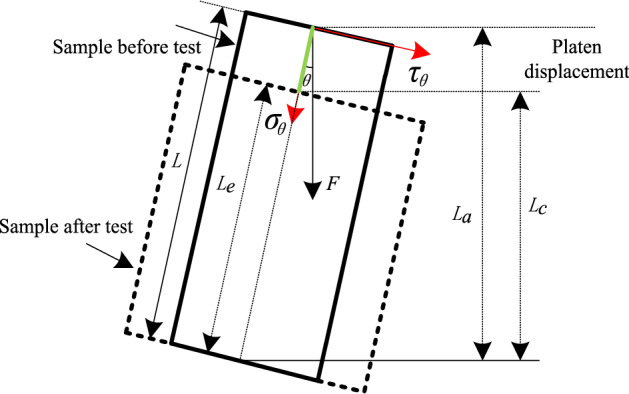
Calculation principle of shear and compression stress components.

Figure 18 presents the granite specimen’s stress and displacement characteristics prior to and following the compression tests. The traditional uniaxial compression test applied the loading force parallel to the specimen surface using a servo-controlled MTS, which is consistent with the 0° granite specimen inclination condition in the C-CAST system. Hence, the specimen axial stress and axial strain are easily obtained as follows^35,51,52^.

11 $$\sigma_{0} = \frac{F}{A}$$

12 $$\varepsilon_{0} = \frac{{L - L_{e} }}{L}$$

where $\sigma_{0}$ and $\varepsilon_{0}$ define the specimen axial stress and axial strain at 0° inclination, respectively; $F$ defines the MTS-exerted axial compressive force on the specimen surface;.$A$ defines the initial cross-sectional specimen area; and *L* and *L*_*e*_ define the initial and final specimen deformation heights, respectively.

At an inclination angle, the vertical loading MTS-applied specimen end face is not presented parallel to the axial specimen direction (Fig. 18). This allowed the decomposition of the total vertical loading into the compression stress component ($\sigma_{\theta }$) perpendicular to the specimen’s end face and shear stress component ($\tau_{\theta }$) parallel to the specimen’s end face. These results nullified Eqs. (11) and (12) for axial stress and axial strain solution determination. As such, the granite specimen’s compression stress, shear stress, and axial strain using the C-CAST system are determined using the following equations, all of which follow the force decomposition principle^26,27^:

13 $$\sigma_{\theta } = \frac{F\cos \theta }{A}$$

14 $$\tau_{\theta } = \frac{F\sin \theta }{A}$$

15 $$\varepsilon_{\theta } = \frac{{L - L_{e} }}{L\cos \theta }$$

where $\varepsilon_{\theta }$ defines the axial strain along the specimen height direction; and $\theta$ defines the specimen inclination angle.
